# Applying Website Rankings to Digital Health Centers in the United States to Assess Public Engagement: Website Usability Study

**DOI:** 10.2196/20721

**Published:** 2021-03-29

**Authors:** Joshua David Calvano, Edwin Lauritz Fundingsland Jr, Deborah Lai, Sara Silacci, Ali S Raja, Shuhan He

**Affiliations:** 1 Department of Research Rocky Vista University College of Osteopathic Medicine Parker, CO United States; 2 Division of Psychology and Language Sciences University College London Bloomsbury United Kingdom; 3 Center for Innovation in Digital HealthCare Massachusetts General Hospital Boston, MA United States; 4 Department of Emergency Medicine Massachusetts General Hospital Boston, MA United States

**Keywords:** website usability, digital health, health care website, usability testing, web interventions, digital health care, web crawler

## Abstract

**Background:**

As the public increasingly uses the internet to search for resources and information regarding health and medicine, it is important that health care organizations provide adequate web resources. Website usability refers to the ease of user experience on a website. In this study, we conducted usability analyses on digital health center websites.

**Objective:**

The primary aims of this study were to (1) replicate a preexisting usability scoring methodology for digital health centers; (2) apply and test this replicated usability scoring methodology on a sample set of digital health center websites; and (3) derive recommendations from the results on potential areas of improvements for our sample of digital health center websites.

**Methods:**

Website usability testing was conducted from March 1, 2020, to March 15, 2020. We replicated a methodology and scoring system from previous literature and applied them to digital health center websites. Our sample included 67 digital health centers that were affiliated with US universities or hospital systems. Usability was split into the following four broad categories: accessibility, marketing, content quality, and technology. Usability tools were used to score websites in each of the four categories. The composite of the key factors of each category was used to generate a general usability and overall usability score for each website.

**Results:**

The category with the highest average score (6.3) was content quality. The content quality score also had the highest SD (2.18) and an SE of 0.27. The lowest performing category was technology, which had an average score of 0.9. The technology score also had the smallest SD (0.07) and an SE of 0.01.

**Conclusions:**

Our data suggest that content quality, on average, was the highest scoring variable among digital health center websites. As content is crucial to digital health knowledge, it is justified that digital health centers invest more resources into creating quality content. The overall lowest scoring variable was technology. Potential reasons for this finding include designated funding for servers, a lack of regulatory frameworks for social media presence and liability, and infrequent website audits. An easy approach for improving this variable is increasing website speed. Accessibility is another area that organizations can potentially improve. We recommend that these organizations perform periodic audits of their web presence with usability tools.

## Introduction

### Background

A hospital’s or digital health care center’s website is often these organizations’ first point of contact with the public; therefore, websites are crucial in first impressions [1,2]. They have the potential to be an important part of the first step in improving patient satisfaction and attracting new patients [3]. In a time when information is expected to be readily available, health care organizations use their websites as key tools for both patient communication and education [4-6]. Patients expect to find current and reliable information on websites that are easily accessible in order to make health-related decisions [7]. As many health-related sources are available (eg, WebMD), health care organizations are aiming to improve their internet presence so that they can better communicate with and market to potential customers [3].

### Website Usability

Improving website usability is a noteworthy approach that medical organizations can use to improve their internet presence, attract and retain more users, and disseminate accurate and reliable information to a larger audience. Usability goes beyond surface-level design; it broadly refers [8] to a product’s user experience, which includes aspects such as the ease of navigation or user-encountered problems within a website [9]. It addresses the question of how easy or pleasing a website is to use, which are factors that can influence the number of users that engage with a website. Usability also addresses users’ level of engagement and a website’s ability to achieve other objectives. When users are not able to easily access and use a website, they are unlikely to continue using it as an information source. Alternatively, improved usability can enhance the reach of a website. It is for this reason that websites are facing the increasing need to conform to user expectations, desires, and requirements [10,11]. Various industries have established standardized guidelines for accessibility, content, marketing, and technology to improve website usability [12-14].

### Usability Studies for Digital Health Centers

Studies have sought to apply usability analyses to e-commerce, e-governments, mobile news apps, and library websites [15-18]. In health care, other studies have analyzed the usability of hospital, children’s hospital, and cancer center websites [3,19,20]. However, to our knowledge, no usability studies have been conducted for digital health centers in the United States. Digital health centers combine innovation-driven health care research with digital technology. Digital health technologies are emerging tools that have the potential to improve patient-centered health care by improving care quality and reducing health care costs [21]. Given digital health centers’ focus on digital technologies (eg, technologies for improving web presence), there is an opportunity to better understand how digital health centers are adapting to technologies that use their web presence. Specifically, we believe that it is distinctly important for these organizations to create websites with high usability to not only improve user experience but also present themselves as leaders in innovation.

### Objectives

The primary aims of this study were (1) to replicate a preexisting usability scoring methodology for digital health centers; (2) to apply and test this replicated usability scoring methodology on a sample set of digital health center websites; and (3) to derive recommendations from the results on potential areas of improvement for our sample of digital health center websites.

## Methods

### Sample Selection

Our focus was on digital health centers that were affiliated with US universities or hospital systems. Indexing the websites of all digital health centers, such as medical companies, was not within the scope of our study.

**Figure 1:**
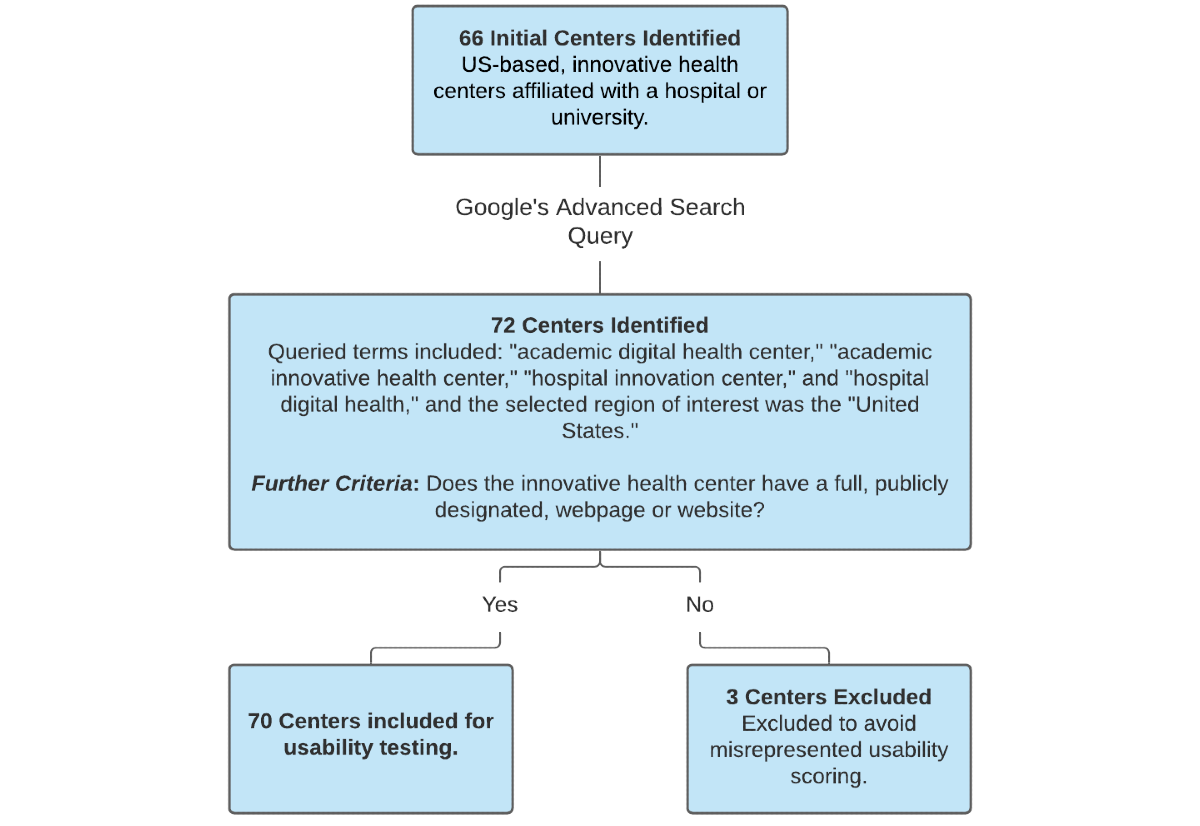
Sample selection criteria for digital health center websites.

The original sample set was derived from Becker’s Hospital Review and consisted of a total of 66 digital health centers [22]. We augmented this sample set by including 8 additional digital health centers that were found with Google’s Advanced Search query builder, which increased the total number of digital health centers in our sample to 72. The terms and phrases searched included “academic digital health center,” “academic innovative health center,” “hospital innovation center,” and “hospital digital health,” and the selected region of interest was the United States. We excluded three digital health centers that did not have a designated digital health center website. Our final sample set consisted of 70 digital health centers (Figure 1).

### Overview

Website usability tests were conducted from March 1, 2020, to March 15, 2020. The methodology used in this study was replicated from previously published health care usability literature [3,19]. We chose to evaluate the same factors as those in the previous studies. However, we modified the definitions for clarity and reproducibility by using our selected assessment tools (Multimedia Appendix 1). We used the same weighted percentages as those from the previous studies and applied specific formulas to these calculated percentages to create a relative scale for comparing usability scores.

In alignment with the replicated methodology, websites were assessed with four scales for the following categories: (1) accessibility, which refers to the ability of people with low levels of computer literacy to access and navigate hospital’s web presence; (2) marketing, which refers to the ability to be found through search engines and the relevance of descriptions to the links provided; (3) content quality, which refers to grammar, the frequency of information updates, material relevancy, and readability; and (4) technology, which refers to download speed, the quality of the programming code, and website infrastructure [3,19]. Each of these categories represent distinct, quantifiable, and actionable areas of usability that digital health centers can improve on to communicate more effectively with their audiences.

### Analysis

All websites were analyzed by using a set of established usability tools (Multimedia Appendix 1). The tools were chosen based on their ability to meet the industry standards for evaluating the selected factors and their relative ease of use. The process for using each tool was based on the tool’s specific instruction manual. One author of this manuscript supervised the training for a team of 6 student reviewers. Each reviewer underwent the same training for using the suite of analytic tools and performing data entry. The reviewers were then directly observed while they used each tool on three websites, in order to confirm proper usage and reliability. Discrepancies and questions were addressed and answered by the supervising author as they arose. In addition, each tool was used on the same local computer to account for irregularities such as differing internet service providers or computing components, which might affect the consistency and reliability of the results. Factors that can vary from second to second, such as speed, were averaged across two separate tools to provide the most accurate values possible.

We built a database of the top-level URLs that were associated with each website in our data set. This was done by using a web crawler, which is a tool that processes URLs and creates topographical maps of a website and all of its subpages. For instance, a top-level domain that corresponds to a website’s home page may be associated with the URL www.healthcare.org. A subpage of this center’s website might be a page about the team members, which might be associated with the URL www.healthcare.org/team. There may be other subpages for specific topics, such as the emergency medicine department and the pediatric department. Once the web crawler creates a topographical map of a website, that website can then be analyzed for page errors, the amount of page content, metadata (ie, titles, keywords, and descriptions), or other preprogrammed factors [23].

Websites in our data set received a final score for the following four categories: accessibility, content, marketing, and technology. Per the replicated methodology, the composite of select key factors across each of the four categories was used to generate a fifth general usability score for each website. A weighted aggregate of these five scores was used as the sixth and final score for the final ranking system.

In the following sections, we describe each of the categories that we evaluated, the development of the rating scale for each of these categories, and the importance of each category’s contributions.

### Accessibility

The accessibility rating indicates a website’s appeal to a broad audience of people with varying literacy levels, technical aptitudes, and disabilities. This category involves factors such as a web page’s meta-description, readability, and the overall layout of the website. A meta-description is the “snippet” page summary that appears in search results when using a search engine. Another factor is functionality, which encompasses elements like actionable buttons that send users to parts of a website and content that is understandable to people with a wide range of education levels or reading abilities. For instance, it has been reported that an estimated 43% of American adults have basic or below basic literacy levels [24]. Accessibility ratings can also be used to evaluate the usability of assistive technologies, such as screen readers and magnifiers for a given website [25]. For our study, we used the Flesch-Kincaid Reading Ease and Gunning Fog Index algorithmic readability scales to rank a website’s reading difficulty and approximate the grade level required to understand each website’s content.

### Content Quality

The content quality rating is used to assess the content on a website. This can include the relevancy of written information to a particular point in time and a specific topic, generated metadata, and the use of a website’s multimedia elements. For instance, a website that is dedicated to supplying information on current closed-loop insulin pumps for people with diabetes may be evaluated on its ability to provide relevant, fact-driven answers to people who seek such information (eg, relative costs, ease of use, etc). In content quality analysis, the multimedia elements on a website may be evaluated for their quality (eg, resolution) and their ability to support the website’s content (ie, available metadata functions). Content quality analysis also involves the assessment of written text (ie, the evaluation of grammar and spelling).

### Marketing

The marketing rating indicates the discoverability of a website. This rating puts particular emphasis on search engine results pages (SERPs), which refer to websites that are suggested to users when they search for information via a web-based search engine, such as Google. Higher placement in search results can lead to greater visibility, and SERPs are considered by some as one of the most important elements of digital marketing. The field of search engine optimization (SEO) involves optimizing a website to achieve higher placement in SERPs, and effectively implementing SEO methods may allow health care organizations to uphold their corporate image as industry leaders [26]. Technical SEO auditing was beyond the scope of this study.

### Technology

The technology rating indicates the technical functionality of a website as opposed to its content quality; it indicates the quality of a website’s technology, technological design, and performance. The technology rating encompasses various aspects, including front-end design, user experience, back-end coding infrastructure, and server management. The front end is what users view when browsing a website. Front-end design involves analyzing HTML elements to ensure that a user is provided with an easily navigable layout and that the website is scalable across devices (ie, computers, mobile phones, and tablets). The back end refers to the programming code upon which the website runs. This code and other website components, such as databases, are stored on servers, which functionally allow people to view websites from their own devices. The servers also affect the speed of the website (eg, how quickly it loads for users), which can play a crucial role in gaining and maintaining users and followers. For instance, a previous study conducted by Google [27] showed that a website that takes longer than 3 seconds to load on a mobile device will lose approximately 53% of its users. Furthermore, the study revealed that the average mobile website speed is upwards of 18 seconds [27].

### General Usability

The general usability rating was based on a composite of select key factors from the prior four categories. The concept of general usability aims to answer the question “how good is my website?” This metric may serve as a starting point for health care organizations to perform an initial audit of their website and identify areas of improvement.

### Overall Usability

An overall usability rank order calculation was performed to comprehensively evaluate all major and minor factors across the five aforementioned categories. Afterward, we assigned weighted percentages to all factors to create an all-inclusive usability ranking system.

## Results

Technical issues prevented the web crawler from running on three websites. This was possibly due to the fact that no index restrictions were set up by the website administrators. We assigned scores to the remaining (N=67) digital health centers.

The subcategory with the highest average score (6.3) was content quality. The content quality score also had the highest SD (2.18) and an SE of 0.27. Accessibility was the second highest scoring subcategory, which had an average score of 2.2. The accessibility score had an SD of 0.51 and an SE of 0.06. Of the four subcategories, marketing had the third highest average score (1.5). The marketing score had an SD of 0.40 and an SE of 0.05. The lowest performing subcategory was technology, which had an average score of 0.9. The technology score also had the smallest SD (0.07) and an SE of 0.01. The summary statistics for all five categories are presented in Table 1.

**Table 1:** Digital health center website summary statistics from the usability analysis.

Category	Score, mean (SE; SD)	Score, range
Accessibility	2.2 (0.06; 0.51)	0.9-3.3
Content quality	6.3 (0.27; 2.18)	1.1-10.7
Marketing	1.5 (0.05; 0.40)	0.6-2.4
Technology	0.9 (0.01; 0.07)	0.7-1.0
General usability	1.5 (0.04; 0.33)	0.8-2.2

The overall rankings for the 67 assessed domains across all categories are reported in Multimedia Appendix 2. The highest scoring centers across all five usability ranking categories were as follows: (1) Sutter Health Design and Innovation (accessibility score=3.3); (2) Sutter Health Design and Innovation (content quality score=10.7); (3) Mayo Clinic Center for Innovation (marketing score=2.4); (4) University of Texas Southwestern Office for Technology Development (technology score=1); and (5) Sutter Health Design and Innovation (general usability score=2.2). In terms of overall usability, the top performing website was that of Sutter Health Design and Innovation (overall usability score=3).

## Discussion

### Comparison With Prior Work

Emerging technologies in the field of digital health are rapidly changing the aspects of health care by making them more patient centered, improving care quality, and decreasing health care costs [21]. The increasing importance of digital health has made it an appropriate field for website usability research.

Our study involved methods that were replicated from previous studies. This allowed us to assess similar trends across various health care website dimensions [3,19]. As with previous studies, the overall scores in our study were highest for the content quality category. This finding could reflect the importance of information to the health care industry and indicate that health centers should invest most heavily in content quality when creating their websites.

Another finding that was consistent with prior research was that the overall lowest ranking category was technology [3,20]. This may be due to a lack of investment in digital technology by the health care industry (eg, investments in server capacity, social media, and website audits). One approach for immediately improving technology is improving website speed. This is largely accomplished through modifying back-end web server settings and minimizing the number of conflicting technologies that run on a website.

A study that evaluated children’s hospitals found accessibility to be the lowest ranking category, which was not the case in our study. However, our accessibility scores were lower than originally anticipated [19]. With regard to health care, we believe that accessibility should be paramount. Health industry leaders should put more effort into ensuring that all domains remain functional and accessible to everyone, so that the quality content on these websites can reach the appropriate users [25].

### Limitations

This study has several limitations. It is common for large organizations to have specific subpages that are dedicated to digital health. For example, one website had an estimated domain age of 33 years when, in reality, the associated innovation health center was aged less than 5 years. However, structuring websites in this manner may provide digital health centers with a competitive advantage, as this method would improve their rankings. This would result in an increase in the number of people who view their website.

Additional limitations and difficulties were found in the assessment of social media websites. Not all social media accounts were directly accessible from these websites. As such, it was difficult to find certain social media accounts through Twitter’s and Facebook’s respective search engines. Oftentimes, the digital health centers’ profiles were distant from the top result.

Assessments of a website’s speed can vary depending on the time of day or the day of data collection. This could be due to changes in the website’s servers, internet connectivity, or computer hardware. To minimize sampling bias, the same computer and the same network were used for all of our tools.

Another limitation was that all information was collected over the course of 2 weeks. As such, several measures may have changed since the initial evaluation.

### Conclusion

With digital health emerging as a leading field in terms of innovation in health care, it is important that digital health centers are able to effectively connect with the public by using their websites. In this study, we conducted an analysis of the overall need for improving the usability of digital health centers’ websites. The average general usability score was 1.5. This shows the necessity of improving the usability aspects of websites. Digital health centers may benefit from taking steps to improve the various components of their websites in order to reach their audiences. A suggested step for these organizations is to perform periodic usability audits of their websites to identify areas for improvement. Several of these institutions have considerable room for improvement in terms of their overall web presence. We have identified approaches that these organizations can use to increase their websites’ usability, such as improving website speed and social media access. These approaches could potentially improve their websites’ reach.

## Appendix

Multimedia Appendix 1 Defined usability factors with their associated percentage weights, assessment tools, impacts, and formulas.

Multimedia Appendix 2 Digital health center websites and category scores.

## Abbreviations

SEO: search engine optimization
SERP: search engine results page
