# Supplementary material for: Applying Website Rankings to Digital Health Centers in the United States to Assess Public Engagement: Website Usability Study
Source: JMIR Hum Factors. 2021 Mar 29;8(1):e20721. doi: 10.2196/20721 (PMC8088849; doi:10.2196/20721)
Supplement: Multimedia Appendix 1 [file humanfactors_v8i1e20721_app1.pdf]

Multimedia Appendix 1: Defined usability factors with their associated percentage weight, assessment tools, impact, and formulas.

Assessment Factors:	Definition:	Accessibility:	Content Quality:	Marketing:	Technology:	General Usability:	Overall Usability:	Assessment Tools[a]:	Applied Formula[b]:
Alternative text	Missing image alternative text. This is used to offer a description of an image file contained on a webpage.	15%		4%	4%	5%	6%	Screaming Frog SEO Spider (Full)	(Total - X) / Total
Amount of content	Assesses the amount of words present on an individual webpage.	8%	33%	9%		5%	9%	Screaming Frog SEO Spider (Full)	Pages >600 / Total
Analytics	Utilizes a Google analytics service for tracking website traffic.			4%	4%	3%	3%	Browser Developer Tools (Free)	Yes/No

Broken Backlinks	Broken inbound backlinks. Backlinks are links from outside websites that link to the website of interest.	8%		1%	8%	5%	4%	AHREFS (Full)	(Relative High - X) / Relative High
Cascading Style Sheets (CSS)	Use of cascading style sheets (CSS) for meeting page design and styling standards.	15%			8%	4%	5%	Pingdom Tools (Free)	Yes/No
Domain age	The age of registered domain name.			4%			1%	GoDaddy WHOIS (Free)	Absolute value
Error page	Assesses for the number of error pages (4xx) within a given website.			4%	8%	3%	3%	Screaming Frog SEO Spider (Full)	(Total - X) / Total
Facebook	Number of Facebook likes for a company's social media page.			8%		4%	3%	Facebook (Free)	X / Relative High
Headings	Number of missing H1 headers on a website.	8%		4%	8%	5%	5%	Screaming Frog SEO Spider (Full)	(Total - X) / Total

In-line CSS	Assesses for the use of embedded CSS throughout the site for additional page formatting.	8%		1%	6%	5%	4%	Pingdom Tools (Free)	Yes/No
Incoming Backlinks	Number of currently functioning backlinks.			9%		5%	4%	AHREFS (Full)	X / Relative High
Meta Data	Number of webpages missing meta descriptions.	4%	13%	7%	4%	5%	6%	Screaming Frog SEO Spider (Full)	(Total - X) / Total
Missing Files	Number of missing structured data files.				8%	3%	2%	Screaming Frog SEO Spider (Full)	(Relative High - X) / Relative High
Open Graph	Use of Facebook's Open Graph protocol			4%	6%	4%	3%	OpenGraphCheck (Free)	Yes/No
Popularity	Alexa Popularity Ranking			9%		6%	4%	Alexa Rankings (Free)	(Relative High - X) / Relative High
Printability	Whether the website utilizes printer-friendly CSS layouts.				8%	5%	3%	Pingdom Tools (Free)	Yes/No

Readability	Assesses the Flesch Kincaid (FK) Reading Ease and Gunning Fog Index (GFI) for reading difficulty and estimated grade level required for understanding.	8%	33%	2%		5%	7%	Readable (Free)	FK 1/2: Absolute Value<br>GFI 1/2:(Relative High - X) / Relative High
Redirections	Number of redirections (3xx) within a website.	6%			6%	1%	2%	Screaming Frog SEO Spider (Full)	(Total - X) / Total
Social Interest	Total number of all social media interest (Twitter and Facebook factors combined).			11%		5%	5%	Facebook and Twitter (Free)	Facebook Likes + Twitter Follows
Speed	The average website speed across multiple assessment tools.	6%		3%	15%	10%	7%	Pingdom Tools and Google Pagespeed Insights (Free)	(Relative High - X) / Relative High
Spelling	Percentage of spelling errors within a given website.		20%				2%	Readable (Full)	100 - X / 100

Twitter	Number of Twitter followers for a company's social media page.			7%		3%	3%	Twitter (Free)	X / Relative High
URL format	Use of URL formats that submit to current SSH protocol.	7%		7%	4%	4%	5%	MOZ URL Structure (Free)	Yes/No
W3C compliance	Number of W3C compliance errors within a website.	8%			8%	5%	4%	W3C Markup Validation Service (Free)	(Relative High - X) / Relative High

[a]Full means that it was the paid, commercially available, version of the product. Free means that it was a free, or open-source.

[b] "X" indicates the obtained value. Relative indicates that the number is relative to the recorded values across the factor.

Note: Percentages do not add to 100 for rounding purposes.
