# Supplementary material for: Applying Website Rankings to Digital Health Centers in the United States to Assess Public Engagement: Website Usability Study
Source: JMIR Hum Factors. 2021 Mar 29;8(1):e20721. doi: 10.2196/20721 (PMC8088849; doi:10.2196/20721)
Supplement: Multimedia Appendix 2 [file humanfactors_v8i1e20721_app2.pdf]

Multimedia Appendix 2: Digital health center websites and category scores

Digital Health Center Website and Link	Accessibility		Content Quality		Marketing		Technology		General Usability		Overall Usability	
	Score	Rank	Score	Rank	Score	Rank	Score	Rank	Score	Rank	Score	Rank
Advocate Cerner Collaborative	2.4	25	7.3	25	1.9	12	0.9	34	1.7	24	2.3	21
Akron (Ohio) Children's Hospital's Center for Patient Experience Innovation	1.6	60	3.7	58	1.5	32	0.8	54	1.1	61	1.5	56
Ascend Innovations	2.6	17	8.5	10	1.1	56	0.8	47	1.8	15	2.3	22
Baylor Scott & White Health's Technology Transfer Office	1.7	54	4.7	51	0.8	65	0.9	39	1.2	52	1.5	58
Baystate Health's TechSpring	2.8	9	9.1	8	1.1	54	0.8	48	1.9	8	2.5	14
Beth Israel Deaconess Medical Center's Technology Ventures Office	2.1	43	5.8	43	1.5	33	1.0	5	1.5	35	2.0	40
Boston Children’s Innovation and Digital Health Accelerator (IDHA)	2.1	38	6.3	36	1.8	13	0.9	27	1.6	29	2.1	29
Brigham and Women's Innovation Hub	2.8	6	9.0	9	1.3	43	0.9	32	2.0	6	2.5	10
Cedar's Sinai Accelerator	2.7	10	8.3	12	1.2	51	0.9	36	1.9	9	2.4	17
Children's Hospital Colorado's Center for Innovation	2.5	22	7.3	26	1.5	29	1.0	2	1.8	18	2.3	20
Children's National Health System's Bear Institute	2.1	41	5.4	47	1.4	40	1.0	4	1.5	42	1.9	43
CHOC Children's Sharon Disney Lund Medical Intelligence and Innovation Institute	2.3	28	7.0	27	1.3	41	0.9	19	1.7	25	2.1	27
Cleveland Clinic Innovations	2.2	33	6.5	32	1.6	24	0.8	45	1.5	33	2.1	33
CU innovations	1.6	57	3.8	56	0.7	66	0.9	30	1.2	56	1.4	63
Dartmouth-Hitchcock Center for Surgical Innovation	1.8	50	4.5	52	1.5	35	0.8	52	1.2	53	1.7	50
El Camino Hospital's Fogarty Institute for Innovation	2.0	48	5.8	44	1.2	47	0.7	66	1.3	48	1.8	48
Florida Hospital's Innovation Lab	2.4	24	7.9	21	0.9	62	0.7	67	1.6	27	2.1	34
Froedtert & the Medical College of Wisconsin's Inception Health	2.7	11	8.1	19	1.0	59	1.0	3	1.9	12	2.3	23

Global Center for Medical Innovation	2.1	40	6.4	33	0.8	64	0.9	29	1.5	36	1.9	45
Hartford (Conn.) Hospital's Center for Education, Simulation and Innovation	2.1	36	6.2	38	1.6	23	0.8	55	1.5	40	2.0	35
Henry Ford Innovation Institute	1.6	58	3.7	59	1.6	26	0.9	18	1.2	57	1.6	54
Holy Name Medical Center Institute for Simulation Learning	2.0	47	6.0	41	0.9	60	0.7	65	1.4	47	1.8	49
Hospital for Special Surgery's Global Innovation Institute	1.4	63	2.9	64	1.6	25	1.0	9	1.1	62	1.4	61
Inspira Health Network Innovation Center	2.5	21	7.6	24	1.1	53	0.8	50	1.7	23	2.2	26
Intermountain Healthcare's Healthcare Transformation Lab	2.0	45	5.2	49	1.5	34	1.0	6	1.4	45	1.9	46
Jefferson Accelerator Zone	1.7	55	4.5	53	1.4	38	0.9	42	1.2	54	1.6	51
Johns Hopkins Medicine's Sibley Innovation Hub	1.6	59	3.5	60	0.9	63	0.9	12	1.2	59	1.4	64
Kaiser Permanente Garfield Innovation Center	2.7	13	8.3	14	1.8	15	0.8	43	1.8	16	2.5	12
Massachusetts General Hospital's John D. Stoeckle Center for Primary Care Innovation	2.2	32	6.4	35	1.7	18	0.8	44	1.5	34	2.1	31
Mayo Clinic Center for Innovation	2.8	8	9.4	6	2.4	1	0.8	58	2.0	5	2.9	2
MD Anderson Innovation Center	2.0	46	5.7	45	1.7	20	0.9	21	1.4	44	2.0	41
MedStar Institute for Innovation	2.6	18	8.2	17	1.9	11	0.8	57	1.8	13	2.5	13
Memorial Sloan Kettering Department of Strategy & Innovation	2.7	12	8.3	13	2.2	3	0.9	13	1.9	11	2.6	5
Montefiore Einstein Center for Innovation in Simulation	1.0	66	1.4	66	1.4	39	0.8	51	0.8	67	1.0	66
Mount Sinai Innovation Partners	2.0	44	6.1	40	1.3	44	0.8	62	1.4	46	1.9	44
Nationwide Children's Hospital's Center for Innovation in Pediatric Practice	1.5	62	2.9	63	1.2	49	0.9	20	1.1	63	1.3	65
Nemours Children's Health System's Center for Healthcare Delivery Innovation	1.7	53	5.0	50	1.7	21	0.9	23	1.3	49	1.8	47
NewYork-Presbyterian Hospitals' NYP Innovation Center	2.1	39	6.1	39	1.6	22	0.8	49	1.5	37	2.0	36

NorthShore University HealthSystem's Grainger Center for Simulation and Innovation	0.9	67	1.1	67	1.4	36	1.0	11	0.8	66	1.0	67
Northwestern Medicine's Center for Primary Care Innovation	2.2	30	6.7	29	1.6	28	0.9	31	1.6	31	2.1	30
NYU Langone Health Tech Hub	2.6	20	8.0	20	2.2	2	0.9	38	1.8	21	2.5	8
Ochsner Health System's Innovation	2.6	15	8.2	16	1.9	10	0.8	56	1.8	20	2.5	11
Orlando (Fla.) Health Strategic Innovations	2.2	34	5.9	42	1.6	27	0.9	17	1.5	41	2.0	38
OSF HealthCare's OSF Innovation	3.0	3	9.5	4	2.0	6	0.9	15	2.1	3	2.8	3
Parkview Health's Mirro Center for Research and Innovation	2.1	42	5.4	46	1.8	16	1.0	7	1.5	43	2.0	37
Penn Medicine Center for Health Care Innovation	1.3	65	2.8	65	1.9	9	0.9	35	1.0	64	1.4	60
Penn State Children's Hospital's Pediatric Innovation Program	1.6	56	3.8	57	0.9	61	0.9	28	1.2	58	1.4	62
ProMedica Innovations	2.2	35	6.4	34	1.7	17	0.9	16	1.5	32	2.1	32
Scripps Translational Science Institute	2.8	7	9.5	5	1.3	42	0.8	61	1.9	10	2.5	9
Spectrum Health Innovations	2.9	5	9.2	7	1.4	37	0.9	22	2.0	7	2.6	6
St. Luke's Technology Ventures	2.9	4	9.6	3	1.1	55	0.9	25	2.0	4	2.6	7
Sutter Health Design and Innovation	3.3	1	10.7	1	2.0	8	1.0	10	2.2	1	3.1	1
The Innovation Institute	2.4	23	7.6	23	1.2	48	0.9	14	1.7	22	2.2	25
The Innovation Studio at Children's Hospital Los Angeles	1.9	49	5.4	48	0.6	67	0.8	59	1.3	50	1.6	52
UCHealth CARE Innovation Center	2.6	16	8.1	18	1.5	31	0.9	41	1.8	19	2.4	16
UNC Center for Health Innovation	2.2	31	6.2	37	2.1	4	0.8	53	1.5	38	2.1	28
UnityPoint Health Innovation Center	3.1	2	10.3	2	1.3	45	0.9	26	2.1	2	2.7	4
University Hospitals' Harrington Discovery Institute	1.8	52	4.3	55	1.2	52	0.8	46	1.2	55	1.6	55
University of California San Francisco's Center for Digital Health Innovation	2.3	29	6.6	31	1.1	57	0.9	24	1.6	30	2.0	39
University of California's Center for Health Quality and Innovation	2.4	27	7.9	22	2.0	7	0.7	64	1.6	26	2.4	18
University of Colorado Hospital's Center for Surgical Innovation	2.1	37	6.6	30	1.2	50	0.8	60	1.5	39	1.9	42

University of Missouri's Tiger Institute for Health Innovation	2.6	14	8.4	11	1.2	46	0.9	37	1.8	17	2.4	19
University of Utah Health Center for Medical Innovation	1.4	64	3.3	61	1.8	14	0.8	63	1.0	65	1.5	59
University of Utah Spark Health Innovation Lab	2.4	26	6.8	28	2.0	5	0.9	40	1.6	28	2.3	24
UPMC Enterprises	2.6	19	8.3	15	1.7	19	0.9	33	1.8	14	2.5	15
UT Southwestern Medical Center Office for Technology Development at BioCenter	1.5	61	3.3	62	1.5	30	1.0	1	1.2	60	1.5	57
Wake Forest Baptist Medical Center's Wake Forest Innovations	1.8	51	4.4	54	1.0	58	1.0	8	1.3	51	1.6	53
